# Carbon Emission Optimization of Ultra-High-Performance Concrete Using Machine Learning Methods

**DOI:** 10.3390/ma17071670

**Published:** 2024-04-05

**Authors:** Min Wang, Mingfeng Du, Yue Jia, Cheng Chang, Shuai Zhou

**Affiliations:** 1 China Merchants Chongqing Communications Technology Research and Design Institute Co., Ltd., Chongqing 400067, China; 2 College of Materials Science and Engineering, Chongqing University, Chongqing 400045, China

**Keywords:** ultra-high-performance concrete, machine learning, artificial neural network, genetic algorithm, carbon emissions

## Abstract

Due to its exceptional qualities, ultra-high-performance concrete (UHPC) has recently become one of the hottest research areas, although the material’s significant carbon emissions go against the current development trend. In order to lower the carbon emissions of UHPC, this study suggests a machine learning-based strategy for optimizing the mix proportion of UHPC. To accomplish this, an artificial neural network (ANN) is initially applied to develop a prediction model for the compressive strength and slump flow of UHPC. Then, a genetic algorithm (GA) is employed to reduce the carbon emissions of UHPC while taking into account the strength, slump flow, component content, component proportion, and absolute volume of UHPC as constraint conditions. The outcome is then supported by the results of the experiments. In comparison to the experimental results, the research findings show that the ANN model has excellent prediction accuracy with an error of less than 10%. The carbon emissions of UHPC are decreased to 688 kg/m^3^ after GA optimization, and the effect of optimization is substantial. The machine learning (ML) model can provide theoretical support for the optimization of various aspects of UHPC.

## 1. Introduction

Ultra-high-performance concrete is a cementitious material with excellent properties. UHPC research has gained popularity recently, and its range of applications has been steadily growing. It has been used in civil engineering including high-rise buildings, bridges, architectural decorating materials, marine structures, explosion-proof structures, nuclear waste storage containers, and thin-walled structures. Materials such as Portland cement, silica fume, mineral powder, fly ash, quartz powder, quartz sand, high-performance water-reduction agent, steel fiber, etc. are the primary types of raw materials adopted in UHPC [1]. Compared to ordinary concrete, the relation between UHPC performance and raw components is more complicated and exhibits a highly nonlinear relationship. The performance of UHPC cannot be predicted using traditional approaches, and obtaining precise regression equations is challenging. High carbon emissions are another major factor restricting the adoption of UHPC. There is presently no uniform design approach or standard for UHPC mix design due to the complexity of raw materials. For designing UHPC mix proportions without adjustment, the conventional mix design procedure for ordinary concrete based on empirical characteristics is not appropriate.

Machine learning (ML) methods have advanced quickly in recent years, and their theories and techniques have been widely utilized to tackle challenging issues in a variety of engineering and scientific domains [2,3,4,5,6,7,8]. Researchers have been driven to apply ANN models and optimization methods to address a variety of civil engineering issues due to the growth of ML techniques [9]. A popular area of research right now is utilizing ANN to predict the mechanical properties of concrete. Ly et al. [10] predicted the compressive strength of self-compacting concrete using the grey wolf optimizer and ANN. Through the application of ANN, Zhu et al. [11] developed a prediction model for the early compressive strength of UHPC. Because ANN is capable of universal approximation, academics have come to value it more and more. The parameters in the model can have an impact on how accurately predictions are made, according to existing studies. The prediction model’s accuracy can be increased, and its physical process can be clarified by utilizing the right parameters. Using ML techniques to improve concrete performance can help avoid its influence mechanism and also offer recommendations for experimental investigation. Sobolev et al. [12] optimized the sequential packaging algorithm using GA and used it to model the tight packing of concrete aggregates. The optimization of concrete mix proportions can be accomplished using this technique. To reduce the risk of early cracking, Chiniforus et al. [13] utilized a mixed design method based on the numerical simulation of concrete heat transfer and GA optimization. Employing the ANN-GA model, Latif et al. [14] optimized the opening ratio and masonry wall stiffness of buildings. Previous research has proved the precision of the ANN-GA model in civil engineering [15].

Due to the enormous number of parameters in UHPC, researchers need to develop novel selection algorithms based on data-driven models. The required model is a function of UHPC components, and by identifying and reducing input parameters, the ANN model can be simplified. It can simultaneously increase computational effectiveness and forecast precision. The UHPC mix proportion with the lowest carbon emission that satisfies engineering requirements can be obtained by creating a concrete performance prediction model that is stressed in engineering, with the required performance indicators as constraint conditions and the carbon emission as the optimization objective.

To achieve ultra-high performance, the usual UHPC compositions employ a lot of cement and other raw materials, which emits 0.68–0.85 tons of CO_2_ per cubic meter of materials [16,17]. Although there are many different paths being developed to improve sustainability in the construction industry, the most practical and feasible ones involve minimizing the amount of Portland cement in concrete by replacing it partially or completely with supplementary cementitious materials (SCMs), such as fly ash (FA), silica fume (SF), and ground granulated blast-furnace slag (GGBFS) [18,19,20,21]. Additionally, maximizing the amount of recycled material in concrete can be achieved by replacing coarse aggregates partially or completely with recycled concrete aggregates, recycled glass, and recycled plastic aggregates [22,23]. These environmentally friendly methods have shown significant potential in reducing embodied carbon. According to Miller et al. [24], the concrete mix ratio also affects the potential for global warming. The life cycle of concrete will be determined by its durability. UHPC is durable and does not even need to be maintained for 80 years [25]. Regarding the application of nano additions, several contributions have noted that employing various cement substitutes could increase the mechanical qualities and durability [26]. Authors also have proved that cementitious materials’ endurance can be further increased by adding SCMs through experiments [27]. Hence, if the carbon emissions can be reduced, the UHPC will have a wider usage. To lower CO_2_ emissions, alternative cement materials should undergo an environmental impact study.

At the beginning of this study, the dataset of UHPC is developed. Then, an ANN prediction model for the 28 day compressive strength and slump flow of UHPC is established. Next, the genetic algorithm is used to optimize the UHPC mix proportion with the lowest carbon emissions, taking into account the minimum 28 day compressive strength and minimum slump flow required in engineering as constraints, as well as the constraints of component content, component proportion, and absolute volume. The experimental results prove the accuracy of the proposed ML model. In order to achieve the lowest carbon emissions while still meeting the engineering specifications for concrete performance, the goal of this research is to simulate the intricate relationship between the composition and performance of UHPC using ML methods. This will increase the likelihood that UHPC will be applied in various applications.

## 2. Machine Learning Methods

### 2.1. ANN

ANN is a research hotspot in the field of artificial intelligence. The development of a nonlinear information processing ANN model involves imitating the neural network of the human brain. A sizable number of nodes (or neurons) coupled to one another make up the model. The activation function, a particular output function, is represented by each node. An artificial neural network’s memory is represented by the connection between each pair of nodes as a weight, which is a value for the signal traveling through the link. The activation function is triggered when a node receives a signal over its threshold, and the outcome is regarded as the input to the following neuron. The network’s activation function, weight value, and connection patterns all influence its output. The connection weight value is *w*_i_. The input *x_i_* is multiplied by *w_i_* on each node. An offset will be included following the addition of all earlier products. The output value is then transferred using the activation function, as seen in Equation (1).

(1) $$y=f\left(\sum_{i=1}^{n}w_{i}x_{i}+Bias_{i}\right)$$

It is capable of accurately approximating any nonlinear function. In order to comprehensively address the issue of hidden layer connection weights in multi-layer neural networks, ANN utilizes the error backpropagation (BP) algorithm [28]. BPNN is a multi-layer feedforward neural network trained using the gradient descent methodology and the error backpropagation algorithm. Between the input layer nodes and the output layer nodes, the ANN inserts numerous hidden layer nodes. Each layer may have multiple nodes. The forward propagation of signals and the backward propagation of errors are two processes that make up the BPNN. While modifying the weights and thresholds is performed in the direction from output to input, calculating the error output is executed in the direction from input to output. The weights and thresholds when the error reaches an acceptable range are established after repeated learning and training. The model has been created and the network training has been finished.

In order to build a BPNN, it is required to decide how many hidden layers there will be and how many nodes will be in each layer. The number of nodes in the input and output layers is equal to the number of input vectors and output vectors, respectively. One can use optimization algorithms or trial-and-error procedures to figure out how many hidden layer nodes there are. By contrasting the network simulation results corresponding to various hidden layer nodes, trial and error methods establish the ideal number of hidden layer nodes. The vast majority of real-world issues can now be resolved by using a single hidden layer, and increasing the number of hidden layers frequently has more drawbacks than the benefits and poorer cost-effectiveness. 

When designing UHPC using the ANN model, the input layer nodes are the UHPC mix ratios, and the output layer nodes are the required performances. While statistical approaches may be impacted by the overfitting of several decision variables and redundant terms in the model, ANN has advantages over traditional statistical methods in modeling a large number of decision variables and objectives. To assure the ANN model’s accuracy, the training process necessitates a wide range of dataset parameters and a substantial amount of data.

### 2.2. Gradient Descent Method

The gradient descent method belongs to an iterative method. The gradient descent approach is frequently used to iterate step by step until the least value of the error function and its accompanying optimal model parameters are obtained while solving model parameters of ML.

The downhill problem can be used to visualize the gradient descent approach. The error function’s initial value is considered to be at a specific peak position, and the iterative process of the error function using the gradient descent method is thought to be a downhill process, or the challenge of going downhill at the fastest speed. The quickest way to get to the bottom of the mountain is to determine the gradient direction at the present location and then move along it with a predetermined step size to reach the next spot. Afterward, the gradient is determined there, the parameters move in the gradient’s direction, and the process is repeated iteratively. When the procedure is finished, the mountain’s bottom will have been reached, signifying that the model’s parameters have been solved and the error function has reached its minimum value.

Stochastic gradient descent (SGD), batch gradient descent (BGD), and mini-batch gradient descent (MBGD) are the three primary variations of the gradient descent method. The randomness in SGD is reflected in the fact that the samples undergo a shuffling operation during each iteration process. After training, the node weights and deviations are updated using a randomly chosen sample. This operation is iterated, and the method does not need to traverse all datasets. BGD needs the calculation of all samples for each iteration, which necessitates the traversing of all datasets to determine errors. As a result, this method demands a heavy computing load and lengthy convergence when the data is enormous. However, BGD can better represent the sample population, and the direction obtained by traversing the dataset is closer to the direction where the extreme value is located. With partial samples used for error calculation and parameter adjustments throughout each iteration, MBGD can be thought of as an intermediate solution between SGD and BGD. Its training speed is significantly faster than that with BGD, and its model convergence is more stable than that with SGD.

### 2.3. GA

GA was first proposed by John Holland in the 1970s, and it was later described as “a kind of simulated evolutionary algorithm” by DeJong and Goldberg et al. [29]. It is a model that simulates Darwin’s biological evolution theory’s natural selection and genetic mechanisms in an effort to find the optimal solution. Six steps make up the fundamental operation procedure of a genetic algorithm.

(1) Initialization: In genetic algorithms, the initial population consists of a set of randomly selected potential solutions. The effectiveness of genetic algorithms is strongly influenced by the initial population. Since the parameters of the solution cannot be handled directly by genetic algorithms, the solved problem must be encoded to become an individual in the genetic space. Binary encoding, octal encoding, hexadecimal encoding, real encoding, tree encoding, permutation encoding, etc. are some of the common encoding techniques. For different types of computational problems, specific forms of transformations play an important role. Although binary encoding can speed up crossover and mutation operations, it is unsuitable for some sophisticated engineering design issues. The use of hexadecimal and octal encoding is not widely used. Real number encoding works better for some more difficult issues, particularly those involving neural networks. Tree encoding can be applied with any encoding language and is appropriate for use in constantly evolving programs or expressions.
(2) Individual evaluation (fitness calculation): The fitness function, which can be single- or multi-objective, is a crucial part of genetic algorithms. The objective function is a function that assesses how well a group of parameters—individuals who make up the population—perform. When creating a fitness function for a practical situation, care should be taken to ensure that it is non-negative, continuous, single-valued, logical, and consistent. Based on specific challenges, the fitness function should be easy to understand and computationally efficient. The iterative convergence of genetic algorithms is directly influenced by the fitness function’s design’s rationality, which also has an impact on the effectiveness of optimization outcomes.
(3) Selection operation: Selection operation controls whether an individual participates in population reproduction, and it also affects how quickly genetic algorithms converge. The Boltzmann algorithm, ranking algorithm, roulette wheel algorithm, random traversal sampling method, etc. are some of the frequently used selection methods. For the selection processes in this study, a roulette wheel algorithm is utilized.
(4) Cross operation: In order to complete the recombination of genetic information and create a new generation of individuals in the population, cross operation simulates chromosomal exchange in biology. The population can be continuously optimized through selection operations when new individuals change in terms of their fitness value. The commonly used crossover operators include single-point crossover, two-point crossover, k-point crossover, uniform crossover, etc.
(5) Mutation operation: Crossover operation produces new individuals with the same alleles as their parents, which will have a detrimental effect on population variety. The goal of mutation operation is to maintain population diversity. The alleles of offspring are changed at random by the mutation operation, and the mutation probability should be as low as feasible or else the genetic algorithm will be identical to the random search algorithm. Exchange mutation, inversion mutation, random shuffling mutation, etc. are some of the frequently utilized mutation operators.
(6) Termination condition judgment: Three criteria need to be met for a genetic algorithm to be judged to have met its termination requirement. The genetic algorithm’s default iteration, which is typically set to 100 to 500, is the first. The genetic algorithm’s iteration process ends, and the ideal individual is produced after the predetermined number of iterations has been reached. The second is to set the fitness function threshold, which allows the genetic algorithm to skip iterations and output the best candidate if an individual satisfies the objective function’s requirements and the required fitness value during the iteration process. The third criterion is that the genetic algorithm has achieved the convergence threshold and cannot achieve the optimization effect through further iterations if the population’s fitness function value is not increasing during the genetic algorithm’s iteration process. As a result, it is possible to finish the iteration and produce the optimal individual.

In this study, the carbon emission function of UHPC is employed as the fitness function, and various UHPC mix ratios serve as various individuals in the population. The individual with the highest fitness is the UHPC mix ratio with the lowest carbon emissions after selection, crossover, and mutation processes.

### 2.4. K-Fold Cross Validation Method

Stone introduced the k-fold cross validation (CV) in 1974, and it is now commonly used in machine learning techniques to choose appropriate hyperparameters [30]. K-fold cross-validation is a commonly used technique for evaluating the performance of machine learning models. Using this approach, the original dataset is divided into k equal subsets. Of these, k-1 subsets are used as training sets, and one subset is used as a validation set. Every k times, this process is performed with a distinct validation set. Lastly, the model’s performance measure is determined by averaging the performance metrics of each validation set. Furthermore, K-fold cross-validation can give a more accurate evaluation of the models’ capacity for prediction while reducing bias and variance issues brought on by incorrectly partitioned datasets. To address the issues of overfitting and underfitting in the training dataset, this work divides the dataset into ten folds [31].

The performance of the prediction model is assessed using the correlation coefficient (R), mean squared error (MSE), and mean absolute error (MAE). The square difference between the predicted and experimental values is calculated via MSE. R determines how closely two variables are correlated. MSE and MAE can measure the predictive ability of the model by calculating the difference between the true and predicted values. MSE, MAE, and R are commonly used indicators to measure the predictive ability of regression models [17,31]. Equations (2)–(4) can be used to calculate *MSE*, *R*, and *MAE*, respectively.

(2) $$MSE=\frac{1}{N}\sum_{i=1}^{N}{\left(y_{i}*-y_{i}\right)}^{2}$$

(3) $$R=\frac{\sum_{i=1}^{N}\left(y_{i}*-\bar{y*}\right)\left(y_{i}-\bar{y}\right)}{\sqrt{\sum_{i=1}^{N}{\left(y_{i}*-\bar{y*}\right)}^{2}}\sqrt{\sum_{i=1}^{N}{\left(y_{i}-\bar{y}\right)}^{2}}}$$

(4) $$MAE=\frac{1}{N}\sum_{i=1}^{N}\left|y_{i}*-y_{i}\right|$$

where y_i_* represents the predicted result and *y_i_* is the experimental result.

## 3. Materials and Experiments

### 3.1. Materials

**Figure 1:**
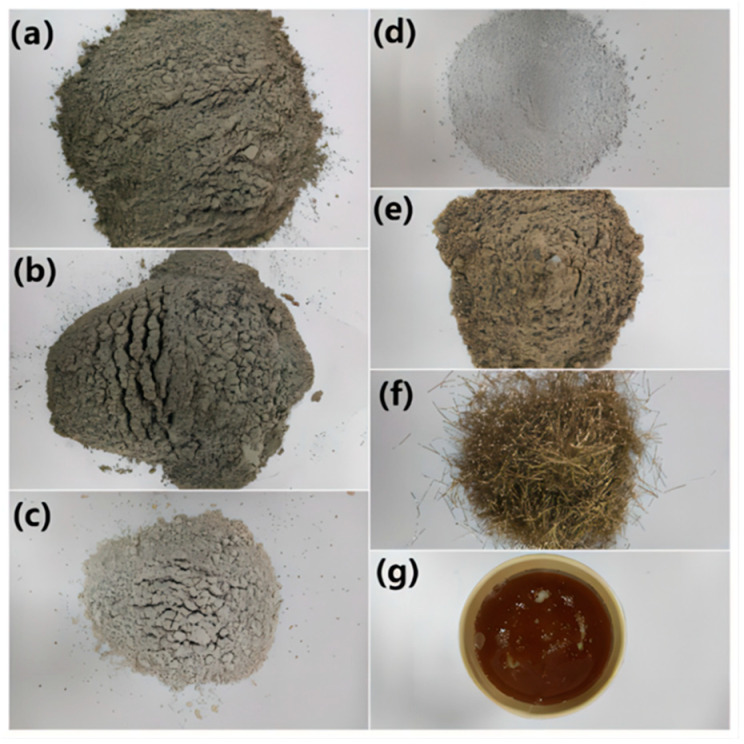
UHPC raw material: (**a**) cement; (**b**) fly ash; (**c**) GGBS; (**d**) silica fume; (**e**) fine aggregate; (**f**) steel fiber; (**g**) superplasticizer.

The raw materials of UHPC in the research contain cement, fly ash (FA), mineral powder (GGBS), silica fume (SF), fine aggregate, steel fibers, superplasticizer, and water, as displayed in Figure 1. Portland cement (P.I. 52.5R) is provided by Sichuan Esheng Company. The Chongqing Fuhuang Company (Chongqing, China) provides first-class FA with a specific surface area of 415 m^2^/kg. Ningxia Boyu Company’s GGBS (Ningxia, China) has a specific surface area of 427 m^2^/kg. SF has an average particle size of 13.3 μm, provided by Shanghai Shanying Environmental Protection Technology Co., Ltd. (Shanghai, China). The chemical makeup and densities of cement, FA, GGBS, and SF are all listed in Table 1. Fine aggregate is made of machine-made sand, with an apparent density of 2630 kg/m^3^ and a fineness modulus of 3.34. The water-reducing agent used in this experiment is a polycarboxylic acid superplasticizer made by China Construction West Construction Co., Ltd. (Chengdu, China), with a water-reducing rate of 45% and a solid content of 40%. The steel fiber is 12 mm long, 0.25 mm in diameter, and 1800 MPa for ultimate tensile strength. 

### 3.2. Experiments

A total of 41 mix proportions are prepared for this experiment, of which 40 are used to confirm the ANN prediction model’s accuracy in predicting UHPC compressive strength and slump flow. The other mix ratio is designed for the validation of the GA optimization of UHPC carbon emissions. The specific mix ratio of UHPC is provided in Table 2. Given that they are supplied for the purpose of testing the ML model, the mix ratio needs to take into account the broadest feasible range. 

First, add cement, fly ash, mineral powder, silica fume, and machine-made sand to the mixer after weighing the UHPC raw materials in accordance with the designed mix ratio. Then, dry mix for 1 min to create a uniform dry mixture. The high-performance water-reducing agent is added to the water, which is then slowly added to the dry mixture while stirring for an additional three minutes. After that, stir the dry mixture for another 4 min while continuing to distribute the steel fibers. For each mix ratio, three sets of 100 mm × 100 mm × 100 mm cube specimens are made after the slump flow of fresh UHPC has been measured. One at a time, pour the mixture into the test mold. To remove bubbles, place the test mold on a vibrating table and vibrate for 30 s. The test mold should be covered with plastic film and left to stand for 24 h at 20 °C ± 2 °C. Finally, leave the UHPC specimen in a standard curing room for 28 days at a temperature of 20 °C ± 2 °C and 95% humidity.

The slump flow test is then conducted. Divided into three layers, the UHPC mixture should be loaded into a slump cylinder after discharge. Use a vibrating rod to equally insert the UHPC mixture 25 times from the edge to the center of each layer. The height of the UHPC mixture sample in each layer after compaction makes up around one-third of the cylinder height. The rod must pierce the whole depth of the slump cylinder while adding the third layer. Once the tamping is finished, take the UHPC mixture out from the cylinder. Vertically raise the slump cylinder. Measure the largest diameter of the mixture expansion surface and its vertical diameter after the UHPC mixture stops diffusing or the diffusion time reaches 50 s. Take the average of the two values as the slump flow when the difference between the two diameter values is less than 50 mm. The slump flow should be remeasured if there is a discrepancy between the two diameter values of more than 50 mm.

**Figure 2:**
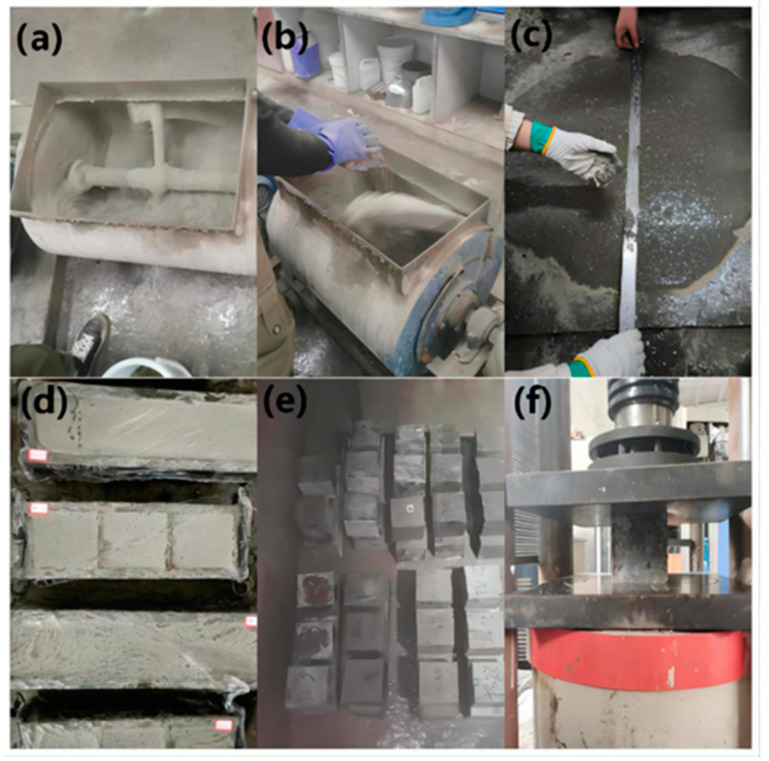
UHPC preparation and test procedures: (**a**) cement, mineral admixture, and sand blending in the concrete mixer; (**b**) adding steel fibers, water, and superplasticizer; (**c**) slump flow tests; (**d**) membrane cover; (**e**) standard curing; (**f**) compressive strength tests.

Compressive strength testing is carried out using a cube specimen having a dimension of 100 mm by 100 mm by 100 mm. When testing compressive strength, a load rate of 12 kN/s is used. The test piece’s surface as well as the upper and lower pressure plates of the testing apparatus should be thoroughly cleaned before the test. The pressure surface should be the side of the UHPC test piece’s forming surface, and the test piece’s center should be lined up with the center of the two plates. The preparation and testing process of UHPC is illustrated in Figure 2.

## 4. Machine Learning Optimization

### 4.1. Database

First, a database for UHPC is built. The raw materials used by UHPC are diverse. Except for the raw materials in the study, some UHPCs contain metakaolin, limestone powder, steel slag powder, rice husk ash, coarse aggregates, and different types of fibers. Considering that there should not be too many types of raw materials used in the production of UHPC in the factory, the study takes into account the most fundamental raw materials. The 28 day compressive strength and slump flow data for UHPC in this study comes from two sources: the first is existing literature [32,33,34,35,36,37,38,39,40,41,42,43,44], and the second is our experimental data, with a total of 422 sets of data gathered. The data from the training group is used to train the ANN, and the data from the testing group is used to test the model. The training group has 261 samples, while the testing group has 160 samples. Together with other experimental results from the literature, the experimental results in the study work as the testing set to test the prediction capacity of the ML model. The mix proportions of each group of specimens are different, and they are also different from those of specimens in other literature. The purpose of adding new samples is to increase the coverage range of the samples, in order to improve the prediction range and accuracy of the model. Meanwhile, 10-fold cross-validation is adopted in the research considering a low number of records. 

Prior to analysis, the input data has to be normalized because the dataset contains varying units and ranges. The collected data are normalized using MinMax to standardize the input variables [45]:

(5) $$x_{norm}=\left(x-x_{min}\right)/\left(x_{max}-x_{min}\right)$$

where *x_min_* and *x_max_* indicate the minimum and maximum values of a certain class of input values, respectively. *x_norm_* indicates the normalized result of a particular class in input values.

### 4.2. ANN Prediction Model

**Figure 3:**
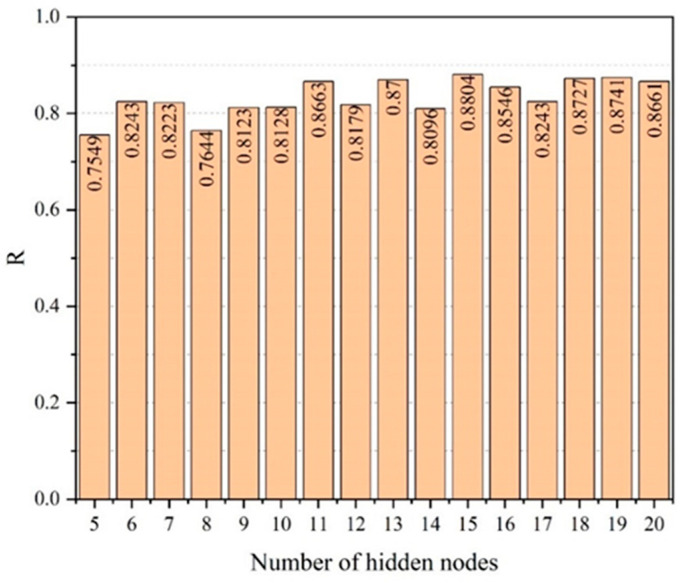
Correlation coefficients R of the ANN model with different hidden nodes.

**Figure 4:**
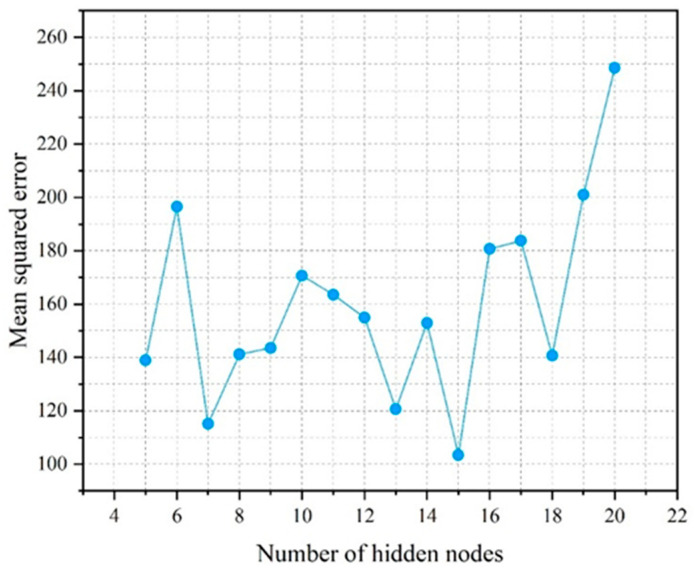
Mean square error of the ANN model with different hidden nodes.

Next, a prediction model for the compressive strength and slump flow of UHPC is developed using ANN. There are eight input layer neurons, which correspond to the content of cement, fly ash, mineral powder, silica fume, fine aggregate, steel fiber, water-reducing agent, and water. There are two output layer neurons which correspond to the compressive strength and the fresh UHPC slump flow. The trial and error method is used to calculate the number of hidden layer neurons. The ANN prediction model’s prediction accuracy is substantially impacted by the number of hidden layer neurons. For each network, networks with five to twenty hidden layer neurons are trained. Ten-fold CV randomly divides the training set into ten parts, selecting one part as the verification set in turn, and the remaining nine parts as the training set. The process is repeated ten times, with different samples as a validation subset. Next, the error is averaged to evaluate the prediction after ten-fold cross-validation is applied. The mean values of the correlation coefficient R and mean square error MSE are then chosen for comparison. Figure 3 and Figure 4 display the outcomes.

**Figure 5:**
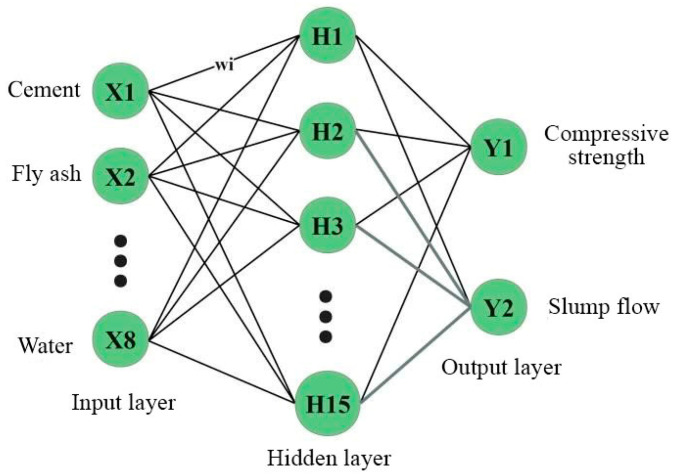
Structure of ANN prediction model for UHPC.

The correlation coefficient R is taken to be 0.8804 when the number of hidden layer neurons is equal to 15, based on the findings in Figure 3 and Figure 4. When there are 15 hidden layer neurons, the MSE likewise falls to its lowest value. Thus, the ANN model with 15 hidden layer neurons exhibits the highest correlation and the least error between the predicted value and the experimental value. For the purposes of developing a prediction model for compressive strength and slump flow, an ANN network with 15 hidden layer neurons is chosen. Figure 5 depicts the structure of the ANN model.

**Figure 6:**
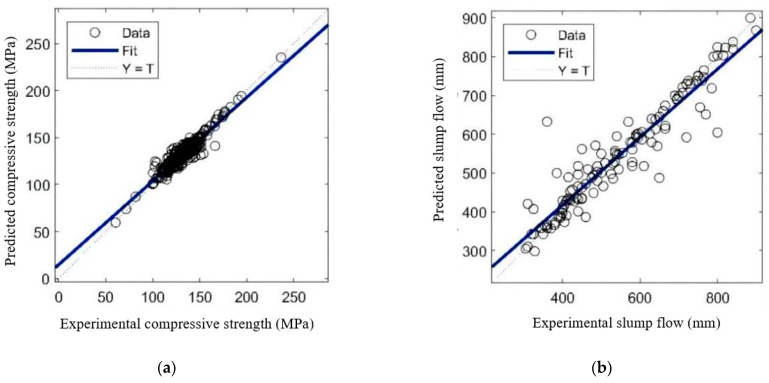
Regression diagram of the training data: (**a**) compressive strength, (**b**) slump flow.

The data are trained multiple times after the ANN’s structure is determined, and the network that performs the best in terms of prediction is chosen for constructing a prediction model for the UHPC 28 day compressive strength and slump flow. Figure 6 exhibits the correlation coefficient R. The compressive strength and slump flow prediction models have R values of 0.94723 and 0.94414, respectively. Both are close to 1, showing that the network’s learning effect is quite strong and that there is a significant connection between the model’s predicted results and the experimental values. The compressive strength and slump flow prediction models have MAE values of 0.7 and 5.79, respectively. The low MAE values also prove the predictive accuracy of the ANN model.

**Figure 7:**
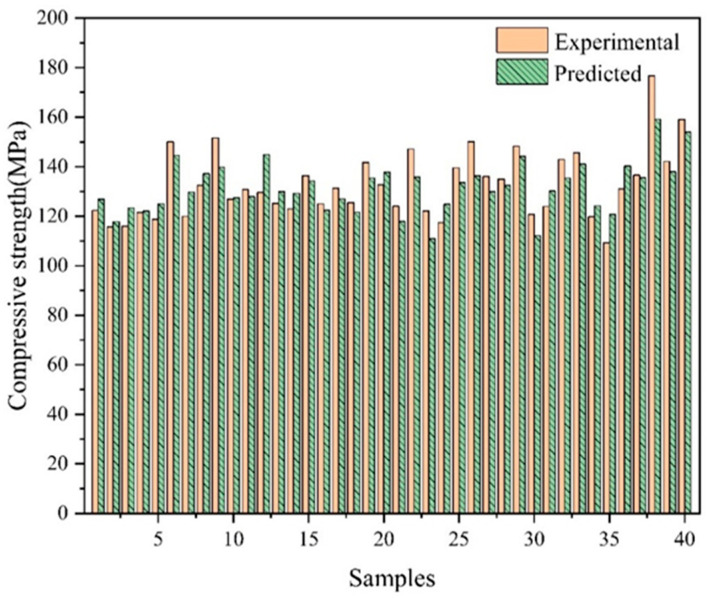
The comparison of compressive strength between the experimental results and predicted results.

**Figure 8:**
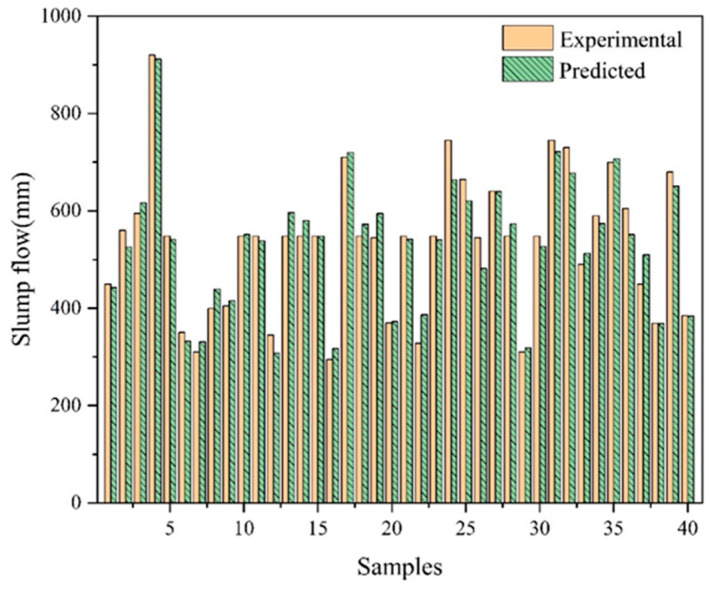
The comparison of slump flow between the experimental results and predicted results.

To predict the 28 day compressive strength and slump flow of UHPC, the trained ANN model is employed. Figure 7 and Figure 8 display the predicted results and contrast them with the experimental values. The prediction error can be used for future samples. The experimental results come from the experiments in Section 3.2. The predicted results are obtained using the ANN model by providing the mix ratio in experiments. With the same mix ratio, the predicted values are typically within 10% of the experimental values. The outcomes of the ANN prediction are good. According to the results, ANN can accurately forecast the compressive strength and slump flow of UHPC. If more pertinent data are gathered, the prediction model’s accuracy will increase even more. Additionally, this demonstrates that machine learning models may be utilized to produce UHPC without requiring numerous trial mixes. 

### 4.3. GA Optimization Process

#### 4.3.1. Calculation Method of Carbon Emissions 

**Figure 9:**
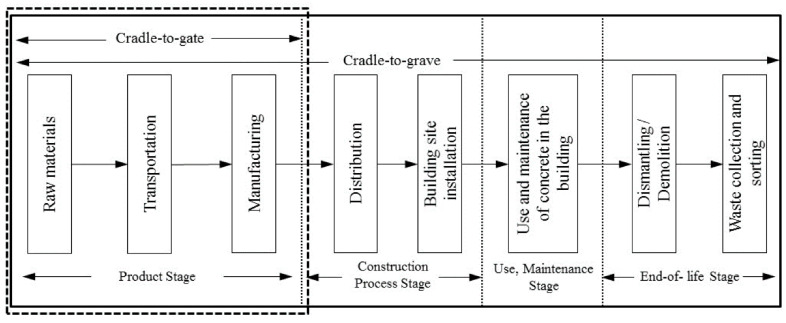
Life cycle assessment (LCA) of UHPC and the boundary in the study.

A life cycle assessment (LCA) is performed to quantify the environmental impacts associated with each mixture design used in this study. The analysis is performed on a declared unit of 1 m^3^ of UHPC for each mixture based on a cradle-to-gate scope. A number of studies have been conducted on LCA calculations to assess the environmental impact of different construction materials [46,47]. The LCA of UHPC is displayed in Figure 9. 

The product stage of concrete (the so-called “cradle to gate” for the product) is selected as a convenient system boundary. UHPC production includes raw material extraction, the transport of the raw materials, and production process. The total CO_2_ emissions can be calculated as follows [48]:

(6) $$CO_{2-e}=CO_{2-eM}+CO_{2-eT}+CO_{2-eP}$$

where *CO_2−e_*, *CO_2−eM_*, *CO_2−eT_*, and *CO_2−eP_* represent total CO_2_ emissions, CO_2_ emissions from raw materials, CO_2_ emissions from transport, and CO_2_ emissions from the mixing operation of UHPC, respectively. 

##### Raw Material Stage

CO_2_ emission from concrete production is calculated as the sum of the quantity of each ingredient used for producing 1 m^3^ of concrete and the CO_2_ emission base units. Equation (7) is used for calculating CO_2_ emission during the production of the raw material required for manufacturing 1 m^3^ of concrete [48]. Table 3 lists the CO_2_ emission reference of each ingredient at the raw material stage. Due to the fact that the volume fraction of steel fibers in the dataset is the ratio of their mass to the total mass of cementitious materials, the two data have been converted.

(7) $$\begin{array}{c} CO_{2-eM}=CO_{2-C}\cdot W_{C}+CO_{2-Fl}\cdot W_{Fl}+CO_{2-GGBS}\cdot W_{GGBS}+CO_{2-Si}\cdot W_{Si}+CO_{2-FA}\cdot W_{FA}\\ +CO_{2-SF}\cdot W_{SF}+CO_{2-SP}\cdot W_{SP}+CO_{2-W}\cdot W_{W} \end{array}$$

Among them, $CO_{2-C},\;CO_{2-Fl},\;CO_{2-GGBS},\;CO_{2-Si},\;CO_{2-FA},\;CO_{2-SF},\;CO_{2-SP},\;\mathrm{and}\;CO_{2-W}$ are the unit mass carbon emissions of cement, fly ash, GGBS, silica fume, fine aggregate, steel fiber, water-reducing agent, and water, respectively. $W_{C},\;W_{Fl},\;W_{GGBS},\;W_{Si},\;W_{FA},\;W_{SF},\;W_{SP},\;\mathrm{and}\;W_{W}$ are the masses of cement, fly ash, GGBS, silica fume, fine aggregate, steel fiber, water-reducing agent, and water in 1 m^3^ UHPC, respectively. $CO_{2-eM}$ is the carbon emissions during the production stage of UHPC.

##### Transportation Stage 

The total quantity consumed and the amount of fuel used for each component are measured in order to determine the CO_2_ emissions at the transportation stage. The distance and method of transport of UHPC ingredients to the manufacturing site are used to determine the CO_2_ emissions. The CO_2_ emission amount for the transportation stage is provided by Equation (8) [46]. The CO_2_ emission reference values for each mode of transportation are listed in Table 4.

*CO_2−eT_* = ∑[(*M(i)/Lt*) × (*d*/*e*) × *CO_2 emission factor T_*] (8)

where $CO_{2-eT}$ is the CO_2_ emissions at the transportation stage for the production of 1 m^3^ UHPC (kg-CO_2_/m^3^); *CO_2 emission factor T_* is the CO_2_ emission factor of the energy resource (kg-CO_2_/kg); *M(i)* is the amount of material used of concrete (kg/m^3^); *Lt* is the transportation load (tons); *d* is the transportation distance (km); and *e* is the fuel efficiency (km/L).

Considering that transportation emissions are highly dependent in all case studies [53,54], this study considers short-distance transportation. The distance of transport of UHPC ingredients to the manufacturing site is taken as 10 km [47,52], and the transport method is by truck.

##### Manufacturing Stage 

The amount of energy used by the equipment required to produce 1 m^3^ of concrete and convert it to CO_2_ can be used to determine the CO_2_ emissions from the production of concrete. The five steps of the concrete manufacturing process are as follows: loading, storing, transporting, measuring for mixing, and mixing. After examining the necessary equipment and data pertaining to power and fossil fuel energy consumed in each stage, the energy required to manufacture 1 m^3^ of concrete can be calculated by calculating the ratio between the capacity of each piece of equipment and the total amount of electricity used. The CO_2_ emission amount for the manufacturing stage is provided by Equation (9) [46].

*CO_2−eP_* = ∑[(*E(i)/R*) × *CO_2 emission factor F_*] (9)

where *CO_2−eP_* is the CO_2_ emission at the manufacturing stage for the production of 1 m^3^ UHPC (kg-CO_2_/m^3^); *CO_2 emission factor F_* is the CO_2_ emission factor (kg-CO_2_/kwh, L, kg)] of an energy resource; *R* denotes the annual UHPC production (m^3^/year)]; and *E(i)* denotes the annual energy usage (unit/year). Here, the *CO_2−eP_* is taken as 7.7 kg-CO_2_/m^3^[47].

#### 4.3.2. Optimization Objective Function

The ANN model is developed for further optimization using the GA method. The compressive strength and slump flow are predicted together in the ANN model for the sake of simplification when conducting the GA optimization process. The UHPC mix proportion optimization can be linked to reducing carbon emissions while satisfying the demands of strength and workability. In essence, the model for predicting compressive strength and slump flow develops a nonlinear functional relationship between UHPC mix proportion and performance. We also need to define an objective function for this optimization, namely the carbon emissions function, which is a linear function, because the optimization aim of this study is the UHPC’s carbon emissions. The carbon emissions function CO_2−e_ is illustrated in Section 4.3.1. The optimal UHPC mixtures show the lowest embodied CO_2_. By adjusting the mix ratio of UHPC, the CO_2_ emissions can be minimized when the strength and workability of UHPC are satisfied. 

#### 4.3.3. Constraint Condition

There are numerous restrictions on how the carbon emissions objective function can be optimized. In this study, the following constraint conditions are taken into account: strength, slump flow, component content, component ratio, and absolute volume.

(1) Compressive strength. The 28 day compressive strength estimated by the ANN model for UHPC should be higher than the strength required. Equation (10) illustrates the strength constraint:
  (10) $$f_{c}\left(28\right)\geq f_{cr}\left(28\right)$$
  Among them, $f_{c}$ (28) is the ANN predicted value of the 28 day compressive strength of UHPC. $f_{cr}$ (28) is the required value for the 28 day compressive strength of UHPC, which needs to be selected according to the requirements in actual engineering. Considering the basic mechanical properties of UHPC, $f_{cr}$ (28) is taken as 120 MPa in this study [55].
(2) Slump flow. The ANN prediction value of the slump flow of fresh UHPC should be higher than the required slump flow. Equation (11) displays the slump flow constraints:
  (11) $$Slump\geq Slump^{r}$$
  where *Slump* is the ANN-predicted value of the workability of fresh UHPC. *Slump^r^* is the required workability of fresh UHPC, which needs to be selected according to the requirements of actual engineering. Considering the basic working performance of UHPC, *Slump^r^* is taken as 600 mm in this study [56].
(3) Component content. The optimized UHPC component content should be within a reasonable range, and this study uses the data range in the dataset as the component content constraint. The component content constraint is shown in Equation (12), and some statistical parameters of the dataset are illustrated in Table 5.
  (12) Lower≤Comp≤Upper
  where *Comp* represents the component content, including cement, fly ash, GGBS, silica fume, fine aggregate, steel fiber, water-reducing agent, and water. *Lower* and *Upper* are the lower and upper limits for each component content.
(4) Component proportion. Some components in UHPC are related, and the proportion of some components should be constrained. This study has considered the water–cement ratio, water–binder ratio, and cement–sand ratio. We still use the proportion range in the dataset as a component proportion constraint. The detailed constraint of composition ratio is shown in Equations (13)–(15). Some statistical parameters of water–cement ratio, water–binder ratio, and cement–sand ratio in the data set are provided in Table 6.
  (13) $${R^{l}}_{w/c}\leq R_{w/c}\leq {R^{u}}_{w/c}$$
  (14) $${R^{l}}_{w/b}\leq R_{w/b}\leq {R^{u}}_{w/b}$$
  (15) $${R^{l}}_{b/fa}\leq R_{b/fa}\leq {R^{u}}_{b/fa}$$
  where *R_w/c_*, *R_w/b_*, and *R_b/fa_* are the water–cement ratio, water–binder ratio and cement–sand ratio, respectively. *R^l^* and *R^u^* are the lower and upper limits of each composition ratio, respectively.
(5) Absolute volume. The absolute volume of UHPC is calculated by Equation (16), which means that the total volume of all components in a 1 m^3^ UHPC should equal 1 m^3^.
  (16) $$\frac{W_{C}}{\rho_{C}}+\frac{W_{Fl}}{\rho_{Fl}}+\frac{W_{GGBS}}{\rho_{GGBS}}+\frac{W_{Si}}{\rho_{Fl}}+\frac{W_{FA}}{\rho_{FA}}+\frac{W_{SF}}{\rho_{SF}}+\frac{W_{SP}}{\rho_{SP}}+\frac{W_{W}}{\rho_{W}}=1$$
  where *W_C_*, *W_Fl_*, *W_GGBS_*, *W_Si_*, *W_FA_*, *W_SF_*, *W_SP_*, and *W_W_* represent the masses of cement, fly ash, GGBS, silica fume, fine aggregate, steel fiber, water-reducing agent, and water in 1 m^3^ UHPC, respectively. *ρ*_C_, *ρ*_Fl_, *ρ*_GGBS_, *ρ*_Si_, *ρ*_FA_, *ρ*_SF_, *ρ*_SP_, and *ρ*_W_ represent the densities of cement, fly ash, GGBS, silica fume, fine aggregate, steel fiber, water-reducing agent, and water, respectively. The densities of cementitious materials are given in Table 1. The densities of fine aggregate, steel fiber, water-reducing agent, and water are 2630 kg/m^3^, 7800 kg/m^3^, 1190 kg/m^3^, and 1000 kg/m^3^, respectively.

#### 4.3.4. Implementation of Carbon Emission Optimization

The UHPC mix ratio can be optimized using GA once the optimization objective function and constraint conditions have been established. The UHPC mix ratio works as the basis for the optimization objective function and the five constraint conditions, which are all applied to the optimization process. As a result, while UHPC progressively lowers its carbon emission through GA, its performance can also satisfy the demands of real-world engineering.

With population size *NP* = 450, maximum iteration count *maxgen* = 200, crossover probability *Pc* = 0.8, and mutation *Pm* = 0.1, carbon emissions are optimized for UHPC by GA. The UHPC carbon emission continuously decreases during the iteration process of the genetic algorithm through initialization, fitness calculation, selection operation, crossover operation, and mutation operation, ultimately obtaining the optimal UHPC mix ratio for carbon emissions.

Due to optimization, the value of the optimization objective function, or the UHPC carbon emission function, continues to drop as the population iterates. The carbon emission function value appears to be mostly constant when the number of iterations approaches 140, demonstrating that the genetic algorithm has reached the convergence threshold. For this investigation, a maximum of 200 iterations are appropriate. The optimized mix ratio for UHPC with the lowest carbon emissions is obtained, and it is displayed in Table 7 as the optimal mix ratio for carbon emissions. This mix ratio has a compressive strength prediction value of 125.1 MPa and a slump flow prediction value of 630.2 mm. The carbon emission CO_2−e_ optimized by GA is 688 kg/m^3^. Compared with the UHPC without ML optimization, the carbon emissions *CO_2−e_* of UHPC with a strength of 120 MPa are 798 kg/m^3^ based on our experiment in Section 3.2. Both results consider the cradle-to-gate boundary and have raw materials with the same embodied carbon. Hence, the results are comparable, and carbon emissions associated with the optimized UHPC mix proportion through GA have significantly dropped.

Finally, the results are further experimentally validated. UHPC specimens are formed according to the optimized low-carbon emission mix ratio and subjected to experimental tests. The compressive strength of UHPC with the optimized mix ratio in Table 7 is 120.5 MPa, with a slump flow of 670 mm through experiments. The error between the experimental values and the ML model is 3.7% and 6.3%, respectively. Meanwhile, the strength and workability constraints are met. It proves that the ML optimization model is suitable for the reduction in carbon emissions.

In this research, the reduction in the carbon footprint is due to the use of a large ratio of by-products. In the concrete industry, the replacement of cement with high-volume SCMs is the most practical and economical way to reduce CO_2_ emissions [57]. Many studies have reduced the CO_2_ emissions of concrete by incorporating mineral admixtures [58]. Our results agree with previous research. 

## 5. Conclusions

The goal of this research is to utilize ML techniques to optimize the UHPC mix ratio and achieve the UHPC mix ratio with the lowest carbon emissions while taking into account the workability and compressive strength of UHPC. The study has a significant impact on encouraging UHPC’s use in engineering.

First, a database on the 28 day compressive strength and slump flow of UHPC is obtained based on previously published research and experimental data on UHPC. Then, utilizing the mix ratio parameters of UHPC as input variables, a prediction model for the 28 day compressive strength and workability of UHPC is developed using BPNN. The strength, workability, component content, component ratio, and absolute volume of UHPC are constraints on the optimization design, with the carbon emissions of UHPC serving as the objective function. By optimizing the objective function through GA, the optimized UHPC carbon emissions are 688 kg/m^3^. The accuracy of the predictions made by BPNN for predicting UHPC performance can be continuously improved by extending the database. Different constraint criteria can be specified using the GA optimization method depending on the various demands for UHPC performance. Results for global optimization with various constraint conditions can be obtained by GA.

## Figures and Tables

**Table 1:** Chemical composition and density of cementitious materials.

	SiO_2_ (%)	Al_2_O_3_ (%)	CaO (%)	MgO (%)	Na_2_O (%)	K_2_O (%)	Fe_2_O_3_ (%)	TiO_2_ (%)	SO_3_ (%)	P_2_O_5_ (%)	Density (kg/m^3^)
P.C	21.39	5.15	61.04	2.82	0.64	0.62	3.86	0.85	3.1	0.10	3190
FA	48.54	27.12	3.19				11.08		1.63		2270
GGBS	26.74	12.36	41.34				4.56		3.89	0.03	2750
SF	94.57	0.67	0.34	0.23		0.82	0.15		2.07	0.90	2310

**Table 2:** The mix proportion of UHPC in the experiments.

Cement/kg	FA/kg	GGBS/kg	SF/kg	Fine Aggregates/kg	Steel Fibers/%	Superplasticizer/%	Water/kg
565	154	154	154	1141	2.0	1.8	164
569	155	155	155	1034	2.0	1.8	165
582	48	194	145	1212	2.0	1.8	165
632	0	0	158	1316	2.0	1.8	223
642	0	0	148	1185	2.0	1.8	158
653	201	0	151	1008	4.0	1.8	161
675	125	0	115	1179	0.0	2.0	180
679	48	97	145	1212	2.0	1.8	155
690	212	0	159	1061	3.0	1.8	191
692	0	0	148	1185	2.0	1.8	158
692	0	99	148	1333	2.0	1.8	134
703	151	0	151	1005	4.0	1.8	161
718	0	0	127	1352	2.0	1.8	152
736	0	0	156	1182	2.0	1.8	173
741	198	0	148	1185	2.0	1.8	158
750	125	0	115	1104	0.0	2.0	180
754	0	157	136	1047	3.0	1.8	209
763	191	0	106	1079	2.0	1.8	173
775	0	161	136	1072	2.0	1.8	193
776	48	0	145	1212	2.0	1.8	165
777	0	0	108	1079	2.0	1.8	173
790	0	105	158	1053	3.0	1.8	189
800	176	0	150	650	2.0	1.8	165
808	0	0	143	1189	2.0	2.0	175
811	0	0	143	1192	2.0	1.8	191
817	0	0	144	1202	2.0	1.8	180
820	0	107	145	1072	2.0	1.8	214
840	0	0	148	1185	2.0	1.8	158
847	0	0	150	997	4.0	1.8	179
850	176	0	150	650	2.0	1.8	165
857	0	0	151	1008	4.0	1.8	191
861	0	0	152	1125	2.0	1.8	202
865	0	54	153	1072	2.0	1.8	193
868	0	0	153	1021	3.0	2.0	183
870	0	0	154	1024	3.0	2.0	189
875	0	0	154	1144	2.0	1.8	206
890	0	0	157	1047	3.0	1.8	209
900	0	0	100	1350	0.4	2.5	170
903	0	0	159	1062	2.0	1.8	204
1000	0	0	0	1350	0.4	2.5	170

**Table 3:** Carbon emissions from raw materials.

Materials	Carbon Emission (kg/ton)	References
P.C	931	[48]
FA	19.6	[48]
GGBS	26.5	[49]
SF	14	[50]
Fine aggregate	1.3	[48]
Steel fiber	1496.5	[51]
Water-reducing agent	250	[48]
Water	0.196	[48]

**Table 4:** Transportation types of concrete ingredients [52].

Type of Transport	Emission Factor kg CO_2_−e t^−1^km^−1^		
Road	0.071		
Rail	0.0166		
Sea	0.0146	-	-

**Table 5:** Statistical parameters of the raw materials.

	Min	Max	Average	Range
Cement (kg/m^3^)	490	1000	754.3	510
FA (kg/m^3^)	0	275	156.8	275
GGBS (kg/m^3^)	0	275	144.6	275
SF (kg/m^3^)	30	210	150.2	180
Fine aggregate (kg/m^3^)	940	1408	1085.7	468
Steel fiber (%)	1	3.5	2.11	2.5
Water-reducing agent (%)	0.4	2.0	1.10	1.6
Water (kg/m^3^)	142	282	185.9	140
Slump flow (mm)	300	920	543.1	620
Compressive strength (MPa)	100.2	190.8	135.7	90.6

**Table 6:** Statistical parameters of *R_w/c_*, *R_w/b_*, and *R_b/fa_*.

	Min	Max	Average	Range
$R_{w/c}$	0.140	0.477	0.239	0.337
$R_{w/b}$	0.120	0.300	0.171	0.180
$R_{b/fa}$	0.65	1.60	1.03	0.95

**Table 7:** Mix proportion for UHPC before and after optimization.

	P.C (kg)	FA (kg)	GGBS (kg)	SF (kg)	Sand (kg)	Steel Fiber (%)	Superplasticizer (%)	Water (kg)	CO_2−eM_ (kg)	CO_2−e_ (kg)
Before optimization	582	145	97	145	1212	0.02	0.01	165	788	798
Optimized	512.4	216.8	180.3	165.5	923.8	1.57	1.65	222.3	678	688

## Data Availability

Data are contained within the article.
